# Pericardial effusion in pediatric paragonimiasis: Surgery may not be necessary in cases of moderate to large effusion—a retrospective study

**DOI:** 10.1371/journal.pntd.0013023

**Published:** 2025-04-24

**Authors:** Houxi Bai, Yanchun Wang, Xiaotao Yang, Yi Huang, Feng Jiao, Yonghan Luo

**Affiliations:** 1 Second Department of Infectious Disease, Kunming Children’s Hospital, Kunming, Yunnan, China; 2 Yunnan Key Specialty of Pediatric Infection (Training and Education Program)/Kunming Key Specialty of Pediatric Infection, Kunming, Yunnan, China; 3 Faculty of Life Science and Technology, Kunming University of Science and Technology, Kunming, Yunnan, China; Cyprus International University: Uluslararasi Kibris Universitesi, CYPRUS

## Abstract

**Background:**

Pericardial effusion is a severe complication of pediatric paragonimiasis, necessitating a careful approach to diagnosis and treatment. Traditionally, the management of pericardial effusion pericardial effusion due to paragonimiasis has involved surgical intervention to drain the accumulated fluid, especially in severe cases. However, the use of non-surgical treatment approaches, such as praziquantel and corticosteroids, have shown promise in certain cases, potentially avoiding the need for invasive procedures.

**Objective:**

To analyze the clinical features and treatment outcomes of pediatric patients with paragonimiasis complicated by moderate to large pericardial effusion, with particular emphasis on comparing surgical and non-surgical treatment approaches, thereby providing clinical evidence for non-surgical treatment in such cases.

**Methods:**

We conducted a retrospective analysis of clinical data from patients diagnosed with paragonimiasis at Kunming Children’s Hospital between January 2014 and April 2023. The patients were divided into pericardial effusion group and thoracopulmonary group. Then the pericardial effusion group was further subdivided into surgical and non-surgical groups. The clinical data were compared between the two groups. Categorical variables were compared using the χ² test, while continuous variables were compared using the t-test or Mann-Whitney U test. Further follow-up was conducted to assess the treatment outcomes in both the surgical and non-surgical groups.

**Results:**

Compared to the thoracopulmonary group (n = 61), patients with pericardial effusion (n = 35) were more likely to present with dyspnea, weak heart sounds, abdominal pain/bloating, and vomiting, and a shorter pre-admission disease course, but significantly longer hospital stays and higher costs. There were no statistically significant differences in clinical presentation, white blood cell count, eosinophil count, pre-admission disease course, or oral praziquantel course between the surgical and non-surgical groups. Notably, pericardial effusion resolved in both groups, but the surgical group had significantly higher hospital costs and longer stays.

**Conclusion:**

In the endemic areas of paragonimiasis,in cases of moderate to severe pericardial effusion a thorough epidemiological history should be taken, and paragonimiasis should be considered. In the absence of life-threatening conditions, early praziquantel treatment, combined with low-dose corticosteroids and regular echocardiography follow-up, can avoid unnecessary surgical intervention if effusion absorption is observed. However, the conclusion of this study is based on limited evidence, and further multicenter, prospective randomized controlled trials are needed to validate the findings.

## Summary

In this study, we examined the response of pediatric patients with paragonimiasis, a parasitic infection, to treatment for moderate to large pericardial effusion, a frequent complication of the disease. Our findings revealed that many children who were treated with oral praziquantel and low-dose corticosteroids experienced complete resolution of the effusion without requiring surgery, contrary to prior recommendations that advocated for surgical intervention in such cases. These results suggest that early non-surgical management can serve as an effective alternative to surgery for children with pericardial effusion due to paragonimiasis, particularly in cases that do not involve life-threatening complications. This study offers valuable new insights into the management of a serious condition, presenting a more cost-effective and less invasive treatment approach.

## Introduction

Paragonimiasis, also known as lung fluke disease (LFD), is a zoonotic foodborne parasitic infection caused by Paragonimus species [1]. Humans primarily contract the disease by consuming raw or undercooked freshwater crabs or crayfish infected with metacercariae, the second intermediate host. The World Health Organization (WHO) ranks paragonimiasis 14th among 24 foodborne parasitic diseases [2], with approximately 195 million people in China at risk of infection annually [3]. The two main pathogenic species in China are Paragonimus westermani and Paragonimus skrjabini. After P. westermani metacercariae enter the human body, they develop into adult flukes in the lungs, causing pulmonary symptoms such as fever, cough, chest pain, and hemoptysis. P. skrjabini larvae can migrate through various organs but rarely develop into adult flukes in humans, leading to ectopic paragonimiasis with symptoms outside the thoracic region [1].

Reports [4,5] suggest that up to 29.3-37.7% of pediatric paragonimiasis cases present with pericardial effusion(PE), but there is limited literature on the management of this complication. Some studies [6,7] indicate that children with PE, compared to those with pleural or peritoneal effusions, are more likely to be misdiagnosed and require longer hospital stays. Wu et al [8]. reviewed 57 cases of pediatric paragonimiasis with PE and concluded that surgical treatment is necessary for moderate to severe effusion. They also recommended extended praziquantel courses for such cases. However, our clinical experience indicates that many children with even large PE recover fully through oral praziquantel treatment combined with low-dose corticosteroids, without requiring surgery. However, to date, no studies have explored the efficacy differences between medical and surgical treatments for moderate to large PE caused by paragonimiasis in children. Therefore, this study retrospectively reviews the clinical characteristics, treatment, and outcomes of pediatric patients with moderate to large PE due to paragonimiasis treated at Kunming Children’s Hospital, providing insight for clinical management.

## Materials and methods

### Ethics statement

Although informed consent was waived due to the retrospective nature of the study, this study was approved by the Ethics Review Committee of Kunming Children’s Hospital. Furthermore, we ensured that all patient data were anonymized to maintain confidentiality and privacy, in accordance with ethical standards. This study was carried out in accordance with the ethical standards of the Declaration of Helsinki.

### Study population

**Fig 1:**
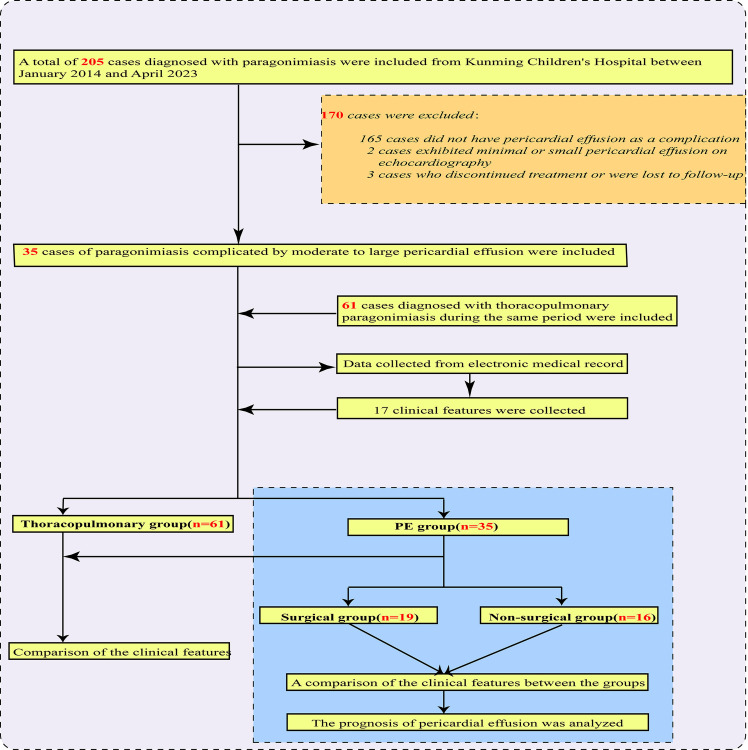
The flowchart of this study.

We retrospectively collected clinical data from 35 pediatric patients diagnosed with paragonimiasis and PE at Kunming Children’s Hospital between January 2014 and April 2023, along with 61 cases of simple thoracopulmonary paragonimiasis. The flowchart of this study is presented in **Fig 1**.

### Inclusion and exclusion criteria

Inclusion criteria: Pediatric patients diagnosed with paragonimiasis according to Chinese health industry standards [9], with moderate to large PE confirmed by echocardiography.

Exclusion criteria: (1) Minimal or small PE on echocardiography; (2) Blood disorders, malignancies, cardiac diseases, metabolic diseases, or non-parasitic PE; (3) Concurrent infections; (4) Patients who discontinued treatment or were lost to follow-up.

### Operational definitions

The criteria for diagnosing PE [8]: Moderate PE (100–500 ml) was defined as a liquid dark area 10–20 mm wide in the pericardial cavity behind the left ventricle on echocardiography, while large effusion (>500 ml) was defined as a liquid dark area >20 mm wide.

The patients were divided into the PE group and the thoracopulmonary group based on the presence of PE. The PE group was further subdivided into surgical and non-surgical groups according to whether they underwent pericardiocentesis or pericardial window operation.

### Criteria for determining patient cure

The criteria for determining patient cure were based on a comprehensive evaluation of clinical symptoms, laboratory parameters, imaging findings, and follow-up assessments. A patient was considered cured upon the complete resolution of symptoms such as fever, cough, chest pain, and dyspnea. Laboratory parameters had to return to normal, including white blood cell and eosinophil counts, along with the reduction of IgG and IgE levels to baseline values. Imaging findings needed to show the resolution of pericardial effusion on echocardiography. Additionally, a follow-up period of at least three months after treatment completion was required to confirm the absence of symptom recurrence or any abnormalities.

### Study variables and data extraction

The baseline characteristics of all hospitalized children were collected, including age, gender, home address, course of disease (defined as the interval duration from the onset of symptoms to hospitalization), clinical outcomes (length of hospital stay, hospitalization costs, and epidemiological history). Symptoms and signs, laboratory test results (including white cell count, eosinophil count, IgG, IgE), imaging findings (chest X-ray, chest CT, cardiac ultrasound, abdominal ultrasound), and oral praziquantel treatment regimens were collected for each group of children.

### Statistical analysis

Statistical analysis was performed using SPSS 26.0 software. Normally distributed data were expressed as mean ± standard deviation (±s), and intergroup comparisons were conducted using the independent samples t-test. Non-normally distributed data were presented as median (interquartile range) [M (P25, P75)] and analyzed using the Mann-Whitney U test for between-group comparisons. Categorical data were described as frequencies and percentages, and the chi-square test was applied. Statistical significance was set at P < 0.05.

## Results

### Clinical features of the pericardial effusion group and thoracopulmonary group

**Fig 2:**
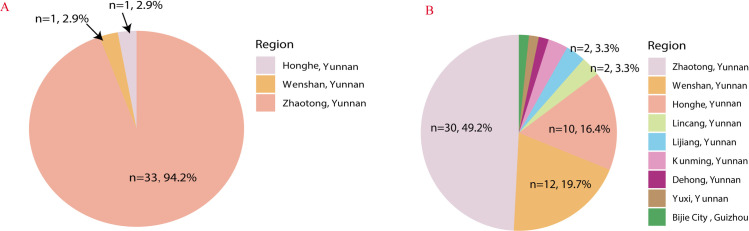
A. Geographical distribution of 35 cases of paragonimiasis with pericardial effusion in children. **B.** Geographical distribution of 61 cases of thoracic pulmonary paragonimiasis in children.

A total of 96 pediatric patients with paragonimiasis were included in this study, of whom 63 (65.6%) were from Zhaotong, Yunnan Province. Thirty-three patients (94.2%) in the PE group (n = 35) and 30 patients (49.2%) in the thoracopulmonary group (n = 61) were from Zhaotong. The remaining 33 cases were from Guizhou, Wenshan, Lijiang, and Honghe **(see** Fig 2). The thoracopulmonary group had a median age of 8.00 [5.92, 9.50] years, consisting of 50 boys (81.97%) and 11 girls (18.03%). Overall, children with paragonimiasis complicated by PE exhibited a higher incidence of dyspnea weak heart sounds compared to those with the thoracopulmonary group. Additionally, gastrointestinal symptoms such as abdominal distension, abdominal pain, and vomiting were more common. These children had a shorter duration of illness prior to admission, longer hospital stays, and higher hospitalization costs (P < 0.05) (**see Table 1**). However, there were no significant differences between the two groups in terms of exposure history, chest pain, cough, hemoptysis, fever, white cell count, or eosinophil count (P > 0.05). IgG testing was performed in 54 children, with 46 (85.2%) showing significantly elevated IgG levels (9.56-61.20 g/L). IgE testing was conducted in 32 children, of whom 29 (90.6%) had markedly elevated levels (370–2188 IU/mL).

**Table 1:** Comparison of clinical characteristics between the pericardial effusion group and the thoracopulmonary group.

	PE group (n = 35)	Thoracopulmonary group (n = 61)	P
Age, Median (Q1, Q3), m	87 [56.50,126.00]	96 [71.00,114.00]	0.626
Sex, n (%)			
Boy	28 (80)	50 (81.97)	0.812
Girl	7 (20)	11 (18.03)	
Contact history			0.063
Raw freshwater	19 (54.29)	19 (31.15)	
Crabs/ Crayfish	3 (8.57)	15(24.59)	
Unknown	5 (14.29)	15(24.59)	
Raw freshwater and Crabs/ Crayfish	8 (22.86)	12 (19.67)	
Chest pain			0.764
Yes	11 (31.43)	21 (34.43)	
No	24 (68.57)	40 (65.57)	
Abdominal pain/bloating			<0.001*
Yes	16 (45.71)	7 (11.48)	
No	19 (54.29)	54 (88.52)	
Vomiting			<0.001*
Yes	11 (31.43)	1 (1.64)	
No	24 (68.57)	60 (98.36)	
Cough			0.072
Yes	17 (48.57)	41 (67.21)	
No	18 (51.42)	20 (32.78)	
Hemoptysis			0.482
Yes	2 (5.71)	6 (9.84)	
No	33 (94.29)	55 (90.16)	
Fever			0.206
Yes	12 (34.29)	29 (47.54)	
No	23 (65.71)	32 (52.46)	
Shortness of breath			<0.001*
Yes	15 (42.86)	7 (11.47)	
No	20 (57.14)	54 (88.52)	
Weak heart sounds			<0.001*
Yes	30 (85.71)	0 (0)	
No	5 (14.29)	61 (100)	
WBC, Median (Q1, Q3),10^9^/L	13.12 [11.61,14.96]	12.36 [9.09,16]	0.213
Eos, Median (Q1, Q3),10^9^/L	2.45 [1.63,4.61]	3.3 [1.15,6.57]	0.870
Course of disease, Median (Q1, Q3), d	7 [5.00,11.50]	20 [10.00,30.00]	<0.001*
Clinical outcomes			
Length of stay, Median, (Q1, Q3), d	13 [9,19]	7 [5,12]	<0.001*
Hospitalization expenses, Median (Q1, Q3), RMB	22104.5 [1144.15,4709.72]	6047.48 [561.10,1320.02]	<0.001*

Abbreviations: PE (pericardial effusion),Q1 (first quartile), Q3 (third quartile), WBC (white blood cell), Eos(eosinophil count),* indicates p < 0.05.

### Characteristics of the surgical and non-surgical groups with pericardial effusion

Based on whether pericardiocentesis or pericardial window operation was performed, the 35 children with paragonimiasis-related PE were divided into the surgical group (n = 19) and the non-surgical group (n = 16). All children in the surgical group underwent pericardial window drainage (n = 18)or pericardiocentesis(n = 1). Pericardial biopsy during surgery revealed chronic granulomatous inflammation with eosinophilia in 5 cases, suppurative inflammation in 7 cases, and acute and chronic inflammation with fibrous connective tissue hyperplasia in 7 cases. There were no significant differences between the surgical and non-surgical groups in terms of age, gender, epidemiological history, clinical symptoms, white cell count, eosinophil count, pre-admission course of illness, or oral praziquantel treatment regimens. However, the length of hospital stays and hospitalization costs were significantly higher in the surgical group (**see Table 2**).

**Table 2:** Comparison of clinical characteristics between the surgical group and the non-surgical group.

	Surgical group (n = 19)	Non-surgical group (n = 16)	P
Age, Median (Q1, Q3), m	90 [79.50,126.00]	73.5 [47.75,122.25]	0.271
Sex, n (%)			0.127
Boy	17 (89.47)	11 (68.75)	
Girl	2 (10.53)	5 (31.25)	
Contact history			0.180
Raw freshwater	13 (68.42)	6 (37.50)	
Crabs/ Crayfish	2 (10.53)	1 (6.25)	
unknown	2 (10.53)	3 (18.75)	
Raw freshwater and Crabs/ Crayfish	2 (10.53)	6 (37.50)	
Chest pain			0.983
Yes	6 (31.58)	5 (31.25)	
No	13 (68.42)	11 (68.75)	
Abdominal pain/bloating			0.067
Yes	6 (31.58)	10 (62.50)	
No	13 (68.42)	6 (37.50)	
Vomiting			
Yes	4 (21.05)	7 (43.75)	0.150
No	15 (78.95)	9 (56.25)	
Cough			0.600
Yes	10 (52.63)	7 (43.75)	
No	9 (47.37)	9 (56.25)	
Hemoptysis			0.202
Yes	0 (0)	2 (12.50)	
No	19 (100)	14 (87.50)	
Fever			0.279
Yes	5 (26.32)	7 (43.75)	
No	14 (73.68)	9 (56.25)	
Shortness of breath			0.352
Yes	10 (52.63)	5 (31.25)	
No	9 (47.37)	11 (68.75)	
Weak heart sounds			0.642
Yes	17 (89.47)	13 (81.25)	
No	2 (10.53)	3 (18.75)	
Pleural effusion			
Yes	18 (94.74)	14 (87.50)	0.446
No	1 (5.26)	2 (12.50)	
WBC, Median (Q1, Q3),10^9^/L	12.56 [10.925,14.42]	14.24 [12.5375,15.645]	0.172
Eos, Median (Q1, Q3),10^9^/L	2.11 [1.6,4.26]	2.86 [2.0025,4.7775]	0.350
Course of disease, Median (Q1, Q3), d	6.50 [4.25,10.00]	7 [5.00,9.50]	0.987
Clinical outcomes			
Length of stay Median, (Q1, Q3), d	16 [12.00,20.50]	10.5[7,16]	0.014*
Hospitalization expenses, Median (Q1, Q3), RMB	29633.5 [24123.08,39200.95]	7776.95 [5784.11,14868.00]	<0.001*
Oral praziquantel course, Median (Q1, Q3), d	2.00 [2.00,3.00]	2.50 [2.00,3.00]	0.806

Abbreviations: Q1 (first quartile), Q3 (third quartile), WBC (white blood cell), Eos(eosinophil count),* indicates p < 0.05.

### Absorption of pericardial effusion in the non-surgical group and surgical group

**Fig 3:**
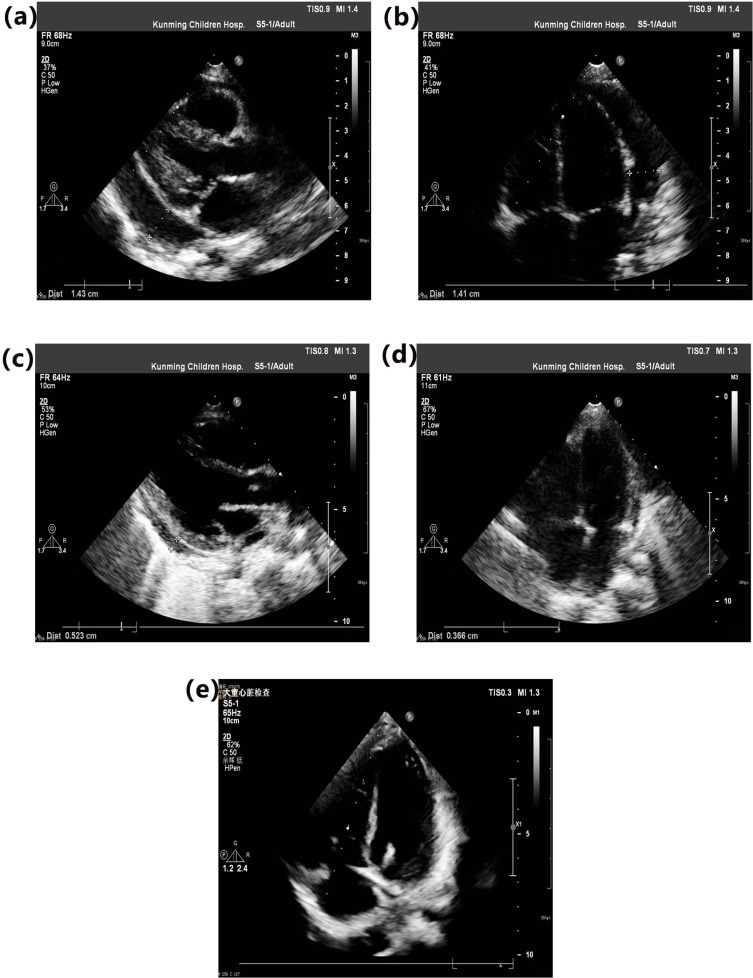
Case 1. **A 9-year-and-4-month-old boy was diagnosed with paragonimiasis accompanied by large pericardial effusion. Following treatment with oral praziquantel and a low dose of corticosteroids, the large pericardial effusion was completely resolved.** (a) Upon admission: Anechoic area in the pericardial cavity detected, posterior left ventricular wall measuring approximately 14.3 mm. (b) Upon admission: Anechoic area in the pericardial cavity detected, lateral left ventricular wall measuring approximately 14.1 mm. (c) After one course of oral praziquantel: Anechoic area in the pericardial cavity detected, posterior left ventricular wall measuring 5.23 mm. (d) After one course of oral praziquantel: Anechoic area in the pericardial cavity detected, lateral left ventricular wall measuring 3.66 mm. (e) After two courses of oral praziquantel: No anechoic area detected in the pericardial cavity.

**Fig 4:**
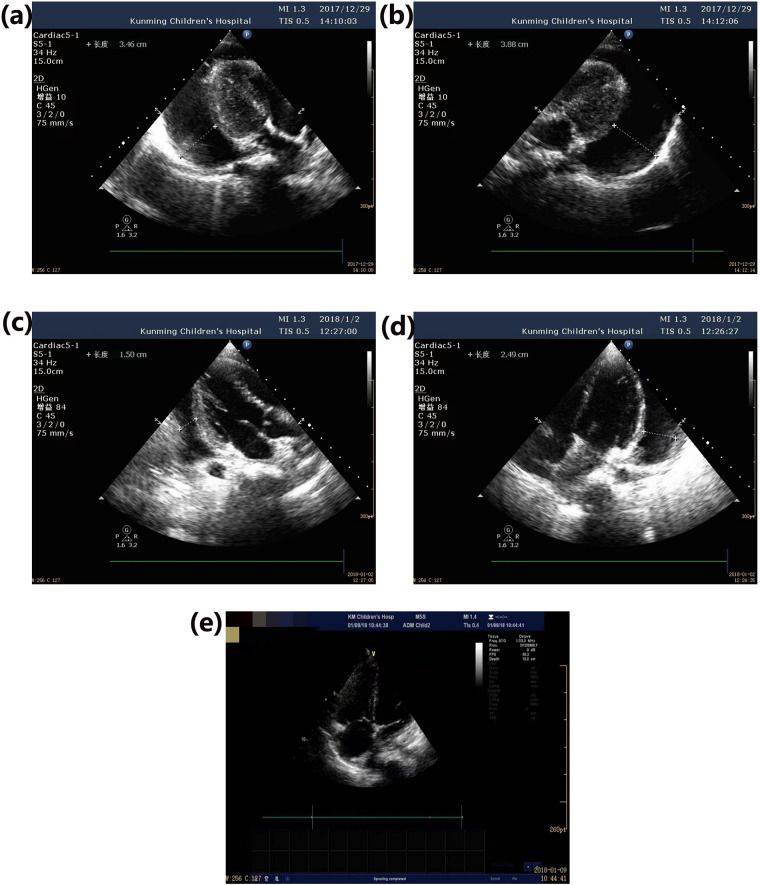
Case 2. **A 6-year-and-10-month-old boy was diagnosed with paragonimiasis accompanied by large pericardial effusion. Following treatment with oral praziquantel and a low dose of corticosteroids, the large pericardial effusion was completely resolved.** (a) Upon admission: Anechoic area in the pericardial cavity detected, posterior left ventricular wall measuring approximately 34.6 mm. (b). Upon admission: Anechoic area in the pericardial cavity detected, lateral left ventricular wall measuring approximately 38.8 mm. (c) After one course of oral praziquantel: Anechoic area in the pericardial cavity detected, posterior left ventricular wall measuring 15.0 mm. (d) After one course of oral praziquantel: Anechoic area in the pericardial cavity detected, lateral left ventricular wall measuring 24.9 mm. (e) After two courses of oral praziquantel: No anechoic area detected in the pericardial cavity.

Sixteen children with non-surgical paragonimiasis-related PE received oral praziquantel treatment at a dosage of 75 mg/kg/day. A complete treatment course was defined as 7 days, with oral praziquantel administered consecutively for the first 3 days, followed by no praziquantel for the remaining 4 days. Additionally, 1 mg/kg/day of oral prednisone acetate was administered concurrently with praziquantel, used only during the first treatment course. After 1–2 courses of oral praziquantel, follow-up cardiac ultrasounds revealed a reduction of moderate-to-large PE to a small amount **(see Figs 3 and 4**). In follow-up blood tests, eosinophil counts decreased in 14 children (87.5%) as the PE diminished. Follow-up over 1–2 months showed complete absorption of the PE (**see Table 3**). In the surgical group, follow-up assessments conducted at 3 days, 7 days, and 2 weeks post-discharge after pericardial window drainage showed no evidence of pericardial effusion.

**Table 3:** Pericardial effusion and eosinophil changes in the nonsurgical group.

Case	Admission to hospital		Praziquantel orally for 1 course		Praziquantel orally for 2 courses		Praziquantel orally for 3 courses	
PE	Eos,10^9^/L	PE	Eos,10^9^/L	PE	Eos,10^9^/L	PE	Eos,10^9^/L
1	Large	1.93	Moderate	0.28	Mild	0.06	/	/
2	Large	2.87	Mild	0.51	No	0.03	/	/
3	Large	5.13	Moderate	2.94	Micro	1.44	/	/
4	Moderate	2.85	Mild	2.84	/	/	/	/
5	Moderate	7.27	Mild	5.64	/	/	/	/
6	Moderate	2.49	No	0.47	/	/	/	/
7	Large	2.10	Mild	1.65	/	/	/	/
8	Large	0.31	Mild	0.75	No	1.06	/	/
9	Large	1.71	Large	1.39	Moderate	0.48	No	
10	Moderate	4.33	Mild	2.45	No	0.23	/	/
11	Large	3.69	Moderate	3.23	Moderate	2.87	No	1.88
12	Large	9.53	Moderate	4.26	Moderate	2.48	Micro	1.12
13	Moderate	1.97	No	0.28	/	/	/	/
14	Large	0.58	Moderate	3.34	Mild	2.14	Micro	0.72
15	Moderate	9.96	No	8.56	/	/	/	/
16	Large	2.14	Mild	0.02	No	0.02	/	/

## Discussion

This study retrospectively analyzed the clinical data of 96 pediatric patients with paragonimiasis, comparing the prognosis of moderate-to-large PE treated surgically and non-surgically. Our study suggests that in some cases of large PE in children with paragonimiasis, oral praziquantel treatment, supplemented by low-dose glucocorticoids, can lead to complete absorption of the PE without the need for surgical intervention. These findings may serve as a reference for clinicians, enhancing their understanding of the disease and potentially reducing unnecessary surgeries.

Paragonimiasis most commonly affects the lungs, causing pulmonary manifestations. However, ectopic paragonimiasis is not uncommon but is easily misdiagnosed. This study found that PE in paragonimiasis is often moderate-to-large. Compared to children with the thoracopulmonary form, those with PE were more likely to experience dyspnea, abdominal pain, and vomiting. These symptoms may result from increased pericardial pressure due to the effusion, leading to impaired ventricular filling during diastole, reduced left ventricular preload, decreased cardiac output, and consequently, dyspnea. Moreover, restricted ventricular diastole and impaired venous return from the inferior vena cava may cause hepatic and intestinal congestion.

The symptoms and signs of paragonimiasis lack specificity, and the disease is frequently misdiagnosed as tuberculosis, fungal infections, malignancies, pyogenic meningitis, or brain tumors, as reported in numerous studies [10–13]. This is particularly true in children who may struggle to accurately describe their symptoms and dietary history, further complicating diagnosis. Misdiagnosis can result in unnecessary medical interventions, imposing significant economic and psychological burdens on patients and their families, while increasing the risk of long-term complications. Our study found that most children with paragonimiasis exhibited marked eosinophilia, highlighting the importance of considering this parameter in routine blood tests in endemic areas. For children with unexplained eosinophilia, a detailed dietary history, including potential consumption of raw water or freshwater crabs/crayfish, should be obtained, supplemented by parasite antibody tests or second-generation sequencing to minimize the misdiagnosis of pediatric paragonimiasis [14].

Southwest China is an endemic area for paragonimiasis, with a high incidence among children. The traditional belief in the region that consuming raw freshwater crabs enhances physical strength (as the crab’s pincers symbolize power) underscores the need for demystification of such beliefs and the promotion of scientific knowledge to aid in disease prevention. Most of the cases in this study were from Zhaotong, where PE was more prevalent, while other regions in Yunnan Province showed a higher proportion of the thoracopulmonary form. This may be related to different subtypes of paragonimiasis endemic to different areas. In Zhaotong, Paragonimus skrjabini predominates, a species that does not mature into adult flukes in the human body, allowing larvae to migrate throughout the body and infect organs other than the thorax and lungs. In contrast, in other regions of Yunnan, Paragonimus heterotremus is more common, a species that matures into adult flukes in the lungs, primarily causing thoracopulmonary disease.

Our study also found that in addition to elevated eosinophil counts, serum IgG and IgE levels were usually significantly increased in children with paragonimiasis, indicating an immune response to parasitic infection. Eosinophils are capable of phagocytizing small parasites, while large parasites require eosinophil-mediated cytotoxicity. Eosinophils can bind to parasitic targets through anti-parasitic IgG and IgE antibodies or CR3b deposited on the parasite’s surface, leading to the parasite’s destruction [15].

Our study indicated that pediatric patients with PE can achieve complete absorption of the effusion without surgical intervention after receiving oral praziquantel combined with low-dose corticosteroid therapy. This finding suggests that, for certain large-scale cases of PE, non-surgical treatment may be a feasible and effective option. This result offered a new perspective for clinical decision-making, especially in cases where there is no life-threatening risk, potentially avoiding unnecessary surgery. When comparing with existing studies, we found that Wu et al [8]. emphasized that surgery is essential for moderate to large pericardial effusions to prevent constrictive pericarditis caused by the viscous nature of the effusion and fibrous exudates. However, our findings differ from this perspective. In our study, patients in the non-surgical group, after receiving praziquantel and corticosteroid treatment, had complete absorption of the pericardial effusion, with no development of constrictive pericarditis during follow-up. This suggests that, while surgery is recommended in many studies, pharmacological treatment may also be effective in certain cases. In these instances, the use of low-dose corticosteroids helps control the inflammatory response and promote the absorption of the pericardial effusion, aligning with the role of corticosteroids in other types of allergic reactions and inflammatory diseases [16].There is limited literature on non-surgical treatment of PE, and a key finding of our study is that corticosteroids play an important role in controlling inflammation and facilitating effusion absorption. Our results also demonstrated that corticosteroids may effectively alleviate the excessive immune response induced by parasitic infections, promoting the resolution of PE. Similar studies have reported the efficacy of corticosteroids in treating other allergic and immune-mediated diseases [17,18].

Our research provided clinical evidence for non-surgical treatment, indicating that oral praziquantel combined with low-dose corticosteroids is effective for moderate to large PE, particularly in non-life-threatening cases. Thus, we proposed that in cases of moderate to large PE without life-threatening complications, early administration of oral praziquantel combined with low-dose corticosteroids should be considered to monitor the absorption of the effusion, rather than immediately resorting to invasive surgical intervention. Moreover, the majority of our patients achieved complete resolution of PE within just 2–3 courses of treatment, without the need for prolonged praziquantel therapy. In cases where 1–2 courses of treatment are ineffective, invasive surgical intervention should then be considered.

The study also has several limitations. Firstly, a retrospective analysis was used, with only 96 cases ultimately included, and only 35 pediatric patients had parasitic infections complicated by PE, resulting in a small sample size. The small sample size may limit the representativeness and generalizability of the findings, thus potentially not fully reflecting the clinical presentation and treatment outcomes of the disease in a larger population. Secondly, the retrospective design is inherently susceptible to selection bias, as treatment protocols were not randomly assigned, which may have influenced the differences observed between treatment groups due to other confounding factors. Furthermore, this study primarily relied on clinical observations of diagnosed patients to conclude that non-surgical treatment could be an effective alternative. However, due to the lack of randomized controlled trials, we cannot exclude the possibility of interference from individual differences and treatment sequencing on the results. Additionally, due to the retrospective nature of this study, we lack long-term follow-up data on patient prognosis and recurrence rates. This limitation prevents us from fully assessing the sustained efficacy of different treatment approaches and the potential for disease recurrence. Future studies should incorporate long-term follow-up and standardized recurrence assessments to provide a more comprehensive understanding of treatment outcomes.

## Conclusion

In the endemic areas of paragonimiasis, in cases of moderate to severe PE, a thorough epidemiological history should be taken, and paragonimiasis should be considered. In the absence of life-threatening conditions, early praziquantel treatment, combined with low-dose corticosteroids and regular echocardiography follow-up, can avoid unnecessary surgical intervention if effusion absorption is observed. However, the conclusion of this study is based on limited evidence, and further multicenter, prospective randomized controlled trials are needed to validate the findings.

## Supporting information

S1 Data The original data used in this study for analysis. (XLSX)
